# Photoluminescence of planar and 3D InGaN/GaN LED structures excited with femtosecond laser pulses close to the damage threshold

**DOI:** 10.1038/s41598-018-29981-8

**Published:** 2018-08-01

**Authors:** Angelina Jaros, Jana Hartmann, Hao Zhou, Barbara Szafranski, Martin Strassburg, Adrian Avramescu, Andreas Waag, Tobias Voss

**Affiliations:** 1 Institute of Semiconductor Technology and Laboratory for Emerging Nanometrology LENA, TU Braunschweig, Hans-Sommer-Str. 66, 38106 Braunschweig, Germany; 2 Epitaxy Competence Center ec2, Hans-Sommer-Straße 66, 38106 Braunschweig, Germany; 3 OSRAM Opto Semiconductors GmbH, Leibnizstr. 4, 93055 Regensburg, Germany

## Abstract

We study the photoluminescence emission from planar and 3D InGaN/GaN LED structures, excited using a femtosecond laser with fluences close to sample’s damage threshold. For a typical laser system consisting of a titanium-sapphire regenerative amplifier, which is pumping an optical parametric amplifier delivering output pulses of a few tens of MW pulse power with ∼100 fs pulse duration, 1 kHz repetition rate and a wavelength of 325 nm, we determine the damage threshold of the InGaN/GaN LEDs to be about 0.05 J/cm^2^. We find that the relative intensity of the GaN photoluminescence (PL) and InGaN PL changes significantly close to the damage threshold. The changes are irreversible once the damage threshold is exceeded. As the damage threshold is approached, the InGaN luminescence band blue-shifts by several tens of meV, which is attributed to band filling effects. The PL decay time reduces substantially, by about 30%, when the excitation energy density is increased by approximately two orders of magnitude. The results are comparable for 2D and 3D LED structures, where in the latter case m-plane QWs exhibit different recombination dynamics because of the absence of the quantum confined Stark effect.

## Introduction

The market for light-emitting diodes (LEDs) continues to grow constantly. State-of-the-art LEDs, with active InGaN layers, now reach external quantum efficiencies of more than 60%^1^. Three-dimensional (3D) core-shell microrod structures are one approach to potentially further increase the efficiency (due to reduced current densities) and reduce the production costs (due to increased light emitting area), as reported in refs.^2–5^. Due to their small footprint on the substrate, they have a low defect density. The active area of the LED structure is increased compared to planar samples because the InGaN quantum wells (QWs) are wrapped around the microrods. Conventional planar quantum wells are c-axis oriented and are influenced by the quantum confined Stark effect (QCSE), resulting in internal electric fields and a separation of electron and hole wave functions^6^. In the 3D structures, whereby the QWs are grown on the nonpolar sidewalls of the GaN cores, the impact of the QCSE is reduced, improving the overlap of the wave functions, resulting in an increased recombination rate^7–9^.

For the optical characterisation of planar LED structures, both time-integrated photoluminescence (PL) measurements, normally performed with continuous wave (cw) lasers, as well as time-resolved measurements, realised with pulsed lasers, are widely used and give valuable complementary information. However, there has been much less research into the optical characterisation of 3D LED structures.

Pulsed lasers are used to assess recombination dynamics. However, the short pulse duration, low repetition rates excitation lasers used for these measurements often emit pulse powers in the range of MW or even higher, which may result in an irreversible modification of the GaN surface^10–12^. Therefore, it is mandatory to study the impact of laser pulses on the integrity of the 3D quantum structures before a detailed characterisation of 3D LEDs, in particular close to the damage threshold.

The investigations presented in our previous work^13^ focused mainly on the temperature dependence of the recombination dynamics of photo-excited electron-hole pairs in similar 3D microrod LED structures.

In this paper, we study how the PL characteristics of 3D core-shell microrods change as a function of the energy density of the excitation pulses, including PL intensity, the intensity ratio of the GaN to the InGaN luminescence bands and the decay dynamics. A 2D InGaN/GaN structure was also measured, for comparison to the 3D structure. Time-integrated and time-resolved PL measurements were performed for a range of excitation pulse energy densities. The PL spectra and their transients were compared for the 3D and planar case. We find a shift of several tens of meV of the InGaN luminescence peak for different excitation energy densities and irreversible changes of the intensity ratio when reaching the damage threshold. We determine the threshold to be about 0.05 J/cm^2^ for our excitation conditions. We also observe a faster recombination rate of the photo-generated electron-hole pairs at higher energy densities. These studies show that for reliable and reproducible measurements of the photoluminescence of InGaN/GaN QW structures the knowledge of the modification threshold and of the used excitation energy density is essential.

## Experiment

The LED structures were grown by metal organic vapour phase epitaxy (MOVPE) on GaN/Sapphire substrates in a 3 × 2″ Thomas Swan Close Coupled Showerhead system. The planar sample consists of an approximately 1.5 *μ*m thick n-GaN buffer layer, grown directly on the substrate, followed by a threefold InGaN/GaN multi quantum well (MQW). The QWs have a thickness of about 3 nm, while the GaN barriers are between 5 nm and 10 nm thick. Mg-doped p-AlGaN (∼20 nm) is used as capping layer and as a barrier for the electrons in the MQW.

**Figure 1:**
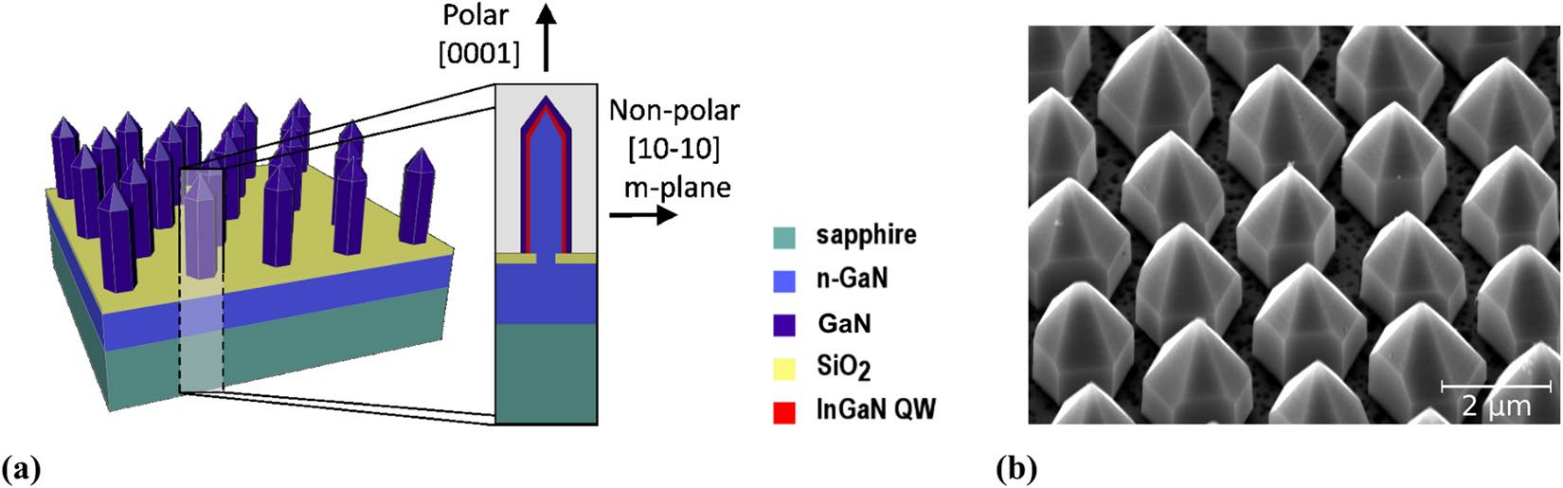
(**a**) Schematic structure of the 3D sample and cross-section of a microrod with core-shell configuration^23^ and (**b**) a SEM image of the investigated 3D sample, tilted by 40°.

The 3D microrod sample was fabricated by continuous selective area MOVPE growth (SAG) through a SiO_*x*_ mask on a GaN/Sapphire substrate previously overgrown with an approximately 4.5 *μ*m thick layer of n-GaN. The core of the rods is n-doped GaN, surrounded by an intrinsic GAN spacer layer and a fivefold InGaN/GaN MQW, which grows as a shell mainly on the non-polar m-plane sidewalls. Finally the MQW is capped with a ∼200 nm thick intrinsic GaN layer. This growth process is described in detail in^14^. The 3D structure of a single microrod is shown schematically in Fig. 1(a), while Fig. 1(b) shows a scanning electron microscopy (SEM) image of the studied sample. We used a 3D structure with an intrinsic GaN capping layer instead of a full LED structure because it showed a high InGaN luminescence, allowing measurements at low excitation energy densities. The diameter of the microrods ranges from 1.7 *μ*m to 1.9 *μ*m, and the height varies between 2.6 *μ*m and 3 *μ*m. Approximately 56% of the substrate surface is covered with microrods.

Continuous excitation, time-integrated PL was measured using a HeCd laser (*λ* = 325 nm, *P*_*out*_ = 20 mW), enabling basic optical characterisation regarding the homogeneity and concentration of Indium in the QWs. Additionally, time-integrated and time-resolved PL was measured under pulsed excitation using a femtosecond laser system (Solstice, Spectra Physics), combined with an optical parametric amplifier (TOPAS-Prime, Light Conversion) with ∼100 fs output pulses at a repetition rate of 1 kHz. The laser spot was focused through a microscope objective, to a spot of ∼5 *μ*m diameter at the sample surface (for both laser systems, excitation in all cases perpendicular to the sample surface). The pulse power used for excitation was varied from 12 W to 46 MW (corresponding to an energy density of 6 *μ*J/cm^2^ to 23 J/cm^2^ per pulse) by using neutral density filters. The measurements were performed at room temperature with *λ* = 325 nm to excite both the GaN layers and the InGaN QWs and to have the same centre wavelength as the HeCd laser. The luminescence was collected with the objective and imaged on the entrance slit of the spectrometer. For the detection of the time-integrated PL, an Acton SP2300 spectrometer (Princeton Instruments) was used, equipped with a liquid-nitrogen cooled CCD camera. A Streak camera (SC-10, Optronis) was attached to the second output port of the same spectrometer for the time-resolved PL measurements.

## Results

**Figure 2:**
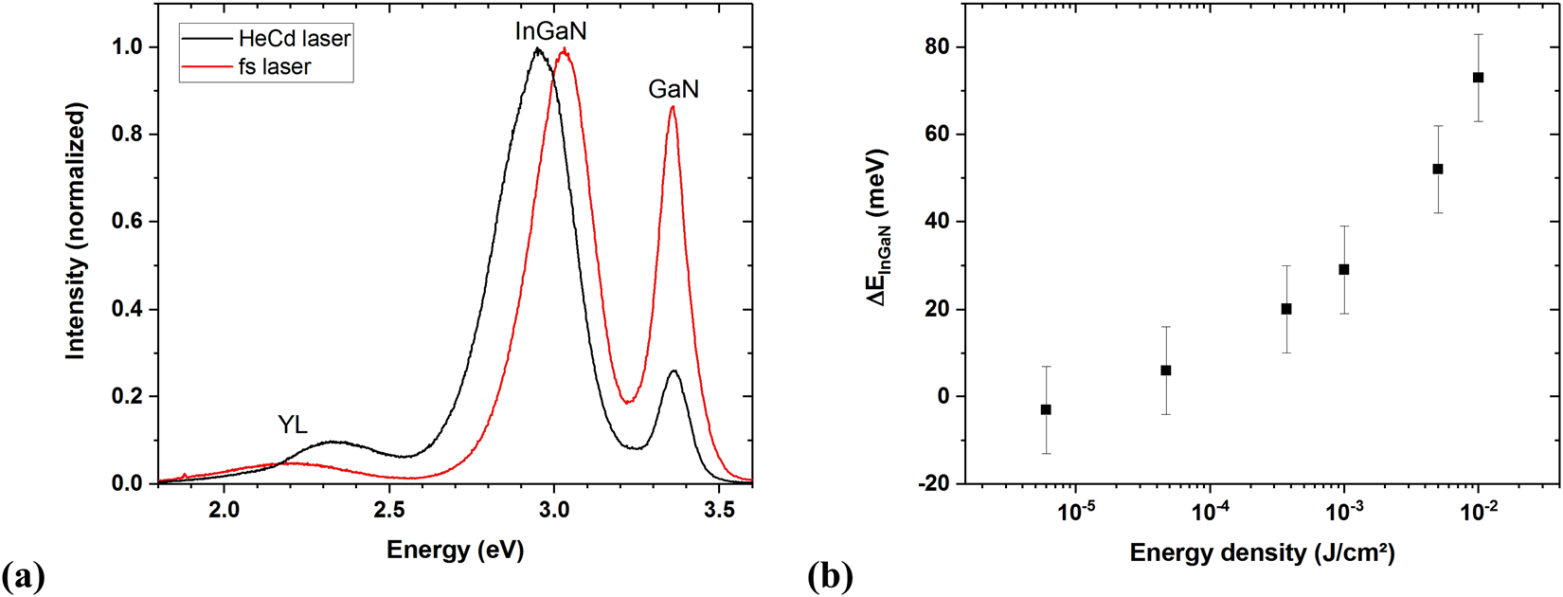
(**a**) Normalized photoluminescence spectra of the 3D LED structure excited by the HeCd laser and the fs laser system with an energy density of 0.01 J/cm^2^ and (**b**) energy difference between the positions of the maximum InGaN intensity under excitation with the HeCd laser and the fs laser.

Two PL spectra of the 3D sample are shown in Fig. 2(a), one using cw excitation (black spectrum) at a power density of 10 kW/cm^2^ and the other using pulsed excitation (red spectrum) with an energy density of 0.01 J/cm^2^. In both spectra, the InGaN, GaN and yellow defect luminescence (YL) bands are clearly visible, with the InGaN luminescence peak being the strongest, centred at 2.96 eV and 3.03 eV for the cw and fs pulsed laser excitation, respectively, a shift of Δ*E*_*InGaN*_ ≈ 70 meV. Both the nonpolar m-plane and semipolar MQWs of the microrords contribute to the observed InGaN luminescence. The GaN PL peak maximum is at 3.36 eV for both measurements. An energy shift of the InGaN peak under different excitation conditions is also observed for the planar sample with Δ*E*_*InGaN*_ in the same order of magnitude. Additionally, the InGaN luminescence band of the planar sample broadened with increasing excitation energy densities (not shown here).

Figure 2(b) shows Δ*E*_*InGaN*_, i.e., the energy position of the InGaN peak emission excited using the fs laser, relative to its energy position when excited using the cw laser, for energy densities between 1⋅10^−2^ J/cm^2^ and 6 ⋅ 10^−6^ J/cm^2^. It should be noted that Δ*E*_*InGaN*_ vanishes within the experimental uncertainty for energy densities in the range of 6 ⋅ 10^−6^ J/cm^2^ to 47 ⋅ 10^−6^ J/cm^2^ and below, which is the lowest energy density used in our experiments.

It has been carefully checked that the peak position of the InGaN luminescence maximum does not change for different power densities of the HeCd laser in the range of 2.4 W/cm^2^ to 10 kW/cm^2^ (constant spot size on the sample).

**Figure 3:**
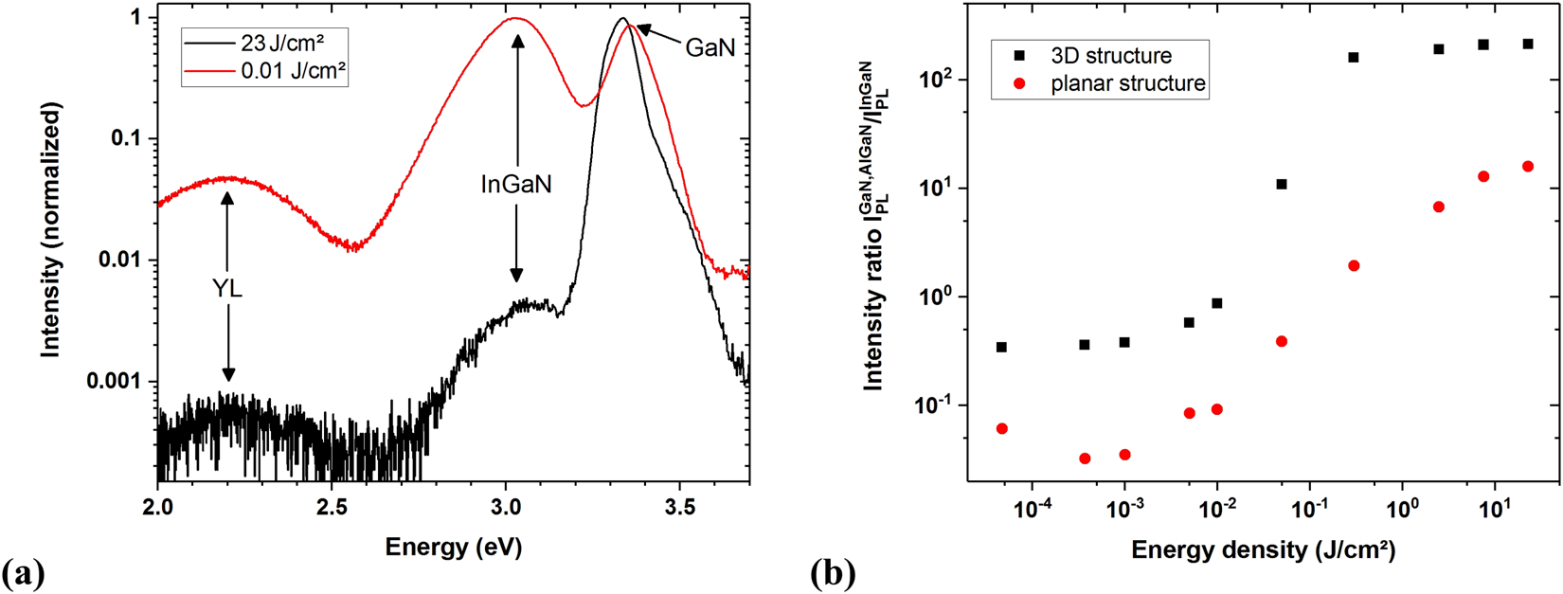
(**a**) Normalized photoluminescence spectra of the 3D LED structure excited by two different laser energy densities and (**b**) intensity ratio *r*(Φ) for the 3D and the planar structure.

Figure 3(a) shows two PL spectra of the 3D structure obtained with the fs laser system as excitation source at 23 J/cm^2^ energy density (black, highest energy density used) and 0.01 J/cm^2^ energy density (red).

In the 0.01 J/cm^2^ PL spectrum (red), the InGaN and GaN luminescence bands are clearly visible with their maxima at 3.03 eV and 3.36 eV, respectively. The yellow defect luminescence band (YL) is located at the low energy side of the InGaN band. The intensity ratio of the maximum GaN PL intensity relative to that of the InGaN PL can be expressed as a function of the energy density Φ as:

1 $$r({\rm{\Phi }})=\frac{{I}^{GaN,AlGaN}({\rm{\Phi }})}{{I}^{InGaN}({\rm{\Phi }})}$$

where *r*(Φ = 0.01 J/cm^2^) = 0.87 (InGaN PL dominating) and *r*(Φ = 23 J/cm^2^) = 212 (GaN PL dominating), respectively.

For the planar structure, a low aluminium content *x* < 10%, was added to the capping layer, so that we compare the intensity of the InGaN layers with that of the Al_*x*_Ga_1−*x*_N in this case. In general, we observed a similar trend: at low energy densities the InGaN PL is prevalent, while for high energy densities the AlGaN PL dominates.

Figure 3(b) shows *r*(Φ) for both samples, showing that it increases by more than two orders of magnitude as the energy density is increased by about seven orders of magnitude. For certain ranges of Φ, *r* can be approximated by:

2 $$r\propto {{\rm{\Phi }}}^{\alpha }$$

As can be seen in Fig. 3(b), there are three regimes with different slopes *α*: for low excitation energy densities up to 5 ⋅ 10^−3^ J/cm^2^, *r* is roughly constant with *α* ≤ 0.1. For 1 ⋅ 10^−2^ J/cm^2^ < Φ < 3 ⋅ 10^−1^ J/cm^2^ *r* increases by 1–2 orders of magnitude with a slope 0.9 ≤*α* ≤ 1.5. At the highest energy densities used, 3 ⋅ 10^−1^ J/cm^2^ < Φ < 23 J/cm^2^, *r* enters into a saturation region. We note that *r* differs by roughly one order of magnitude between the 3D and the planar sample, which is assumed to be a result of the different sample geometries. In particular, the 3D structure has a higher GaN luminescence intensity compared with the AlGaN intensity of the planar sample due to the approximately 200 nm thick GaN capping layer on the rods and the additional GaN on the substrate (i.e., between the rods).

In order to understand if the changes of the intensity ratio of the GaN and InGaN luminescence bands are reversible, i.e., if *r* reaches its previous values when decreasing the energy density again, measurements were performed as follows: First, a PL spectrum was measured with an excitation energy density of 0.01 J/cm^2^ and the intensity ratio was calculated. Afterwards, the energy density was raised for several seconds to an initial value of 0.05 J/cm^2^, before it was decreased to 0.01 J/cm^2^, and once again the intensity ratio was determined from a spectrum taken at this excitation energy density value. This procedure was repeated, incrementally increasing the higher energy density value, following the values listed in Table 1. The intensity ratio *r* obtained at the lower fixed energy density value (Φ = 0.01 J/cm^2^) is given in Table 1. All measurements were performed on the same spot on each sample.

**Table 1:** Intensity ratio *r* (Φ = 0.01 J/cm^2^) for both samples after excitation with different maximum energy densities.

Max. energy density (J/cm^2^)	r(Φ = 0.01 J/cm^2^) 3D structure	r(Φ = 0.01 J/cm^2^) planar structure
0.01	0.93	0.09
0.05	0.84	0.10
0.3	0.76	0.31
2.5	0.81	1.73
7.6	0.56	—
23	0.58	—

For both samples, the PL intensity measured at 0.01 J/cm^2^ started to decrease when the sample had been previously excited with energy densities above 0.05 J/cm^2^. We note that the different PL bands do not decrease equally in both cases. In the case of the 3D structure, the reduction of the GaN and InGaN PL intensities is similar. For the planar structure, there is a significant change in the intensity ratio after the maximum energy density was increased to 2.5 J/cm^2^. Afterwards, the AlGaN PL dominates the spectrum. A missing value in Table 1 indicates that the intensity of the luminescence was too weak to be detected by our measurement system.

**Figure 4:**
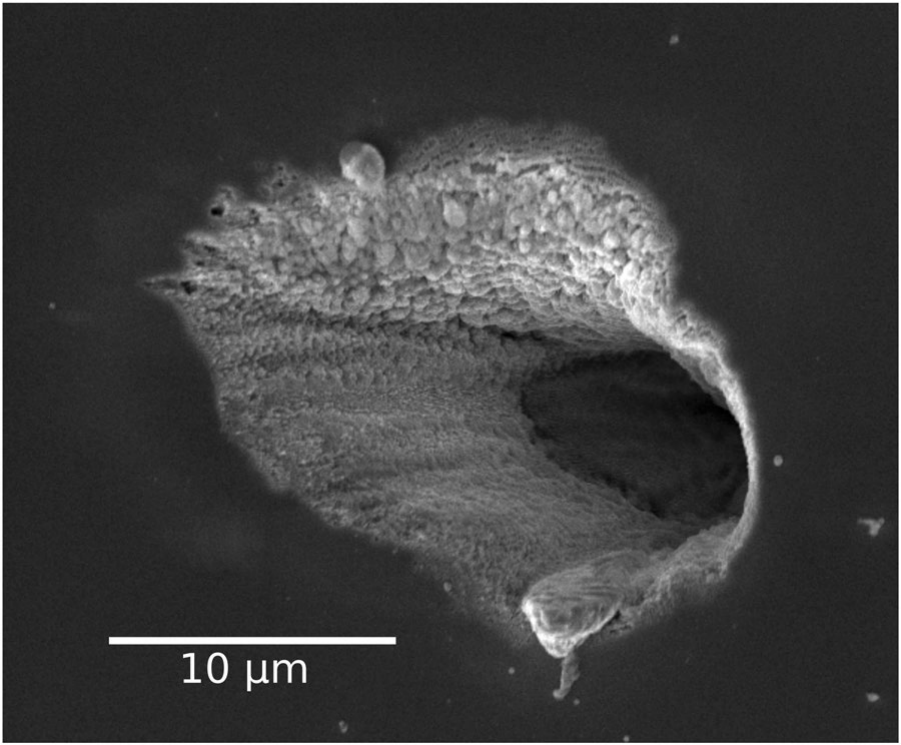
GaN layer after excitation with fs laser pulses for 1 minute with an energy density of 23 J/cm^2^.

To further investigate the influence of the high power laser pulses, a planar sample with an unintentionally doped GaN layer of 11 *μ*m thickness was irradiated with laser pulses with energy densities between 0.01 J/cm^2^ and 23 J/cm^2^. The irradiation time was also varied, from less than a second up to ten minutes. Figure 4 shows an SEM (scanning electron microscope) image of a hole in the GaN layer after an irradiation with the fs laser for one minute with 23 J/cm^2^ energy density.

Due to the irradiation with the high power laser pulses, the GaN was ablated down to the substrate. The extension to the left side is caused by a non-perpendicular incidence angle. We observed modifications of the GaN layer in the form of a contrast difference in the SEM image down to an energy density of 0.05 J/cm^2^ after a treatment of about 10 minutes.

**Figure 5:**
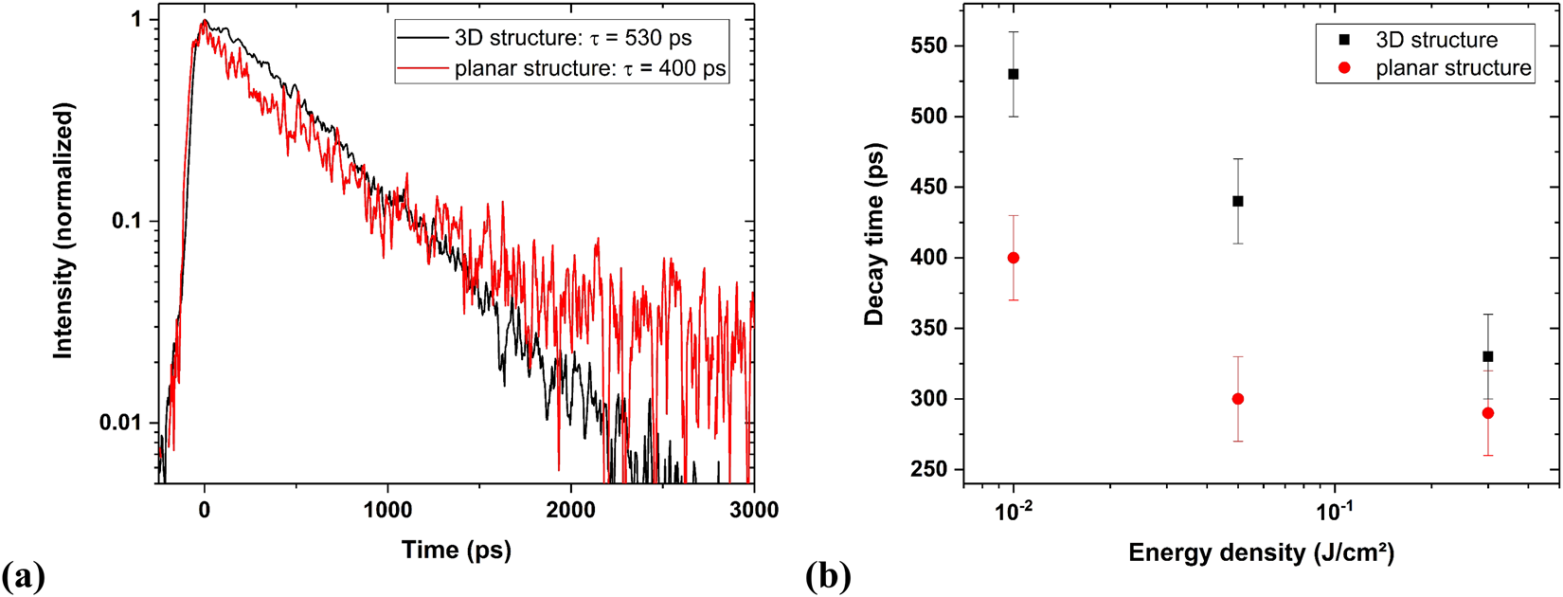
(**a**) InGaN PL transients of the 3D structure (black) and the planar structure (red) excited with 0.01 J/cm^2^ energy density and (**b**) decay times of the 3D structure and the initial decay times of the planar structure for different energy densities.

Time-resolved studies were performed, in addition to the time-integrated PL measurements, in order to characterise the PL decay dynamics of the 2D and 3D structures. Figure 5(a) shows the InGaN PL intensity as a function of time for the 3D (black) and the planar structure (red), using an energy density of 0.01 J/cm^2^. For both transients, the intensity of the InGaN peak has been integrated over the energy range around the maximum where *I* > *I*_*max*_/*e*.

Immediately after the maximum intensity has been reached at *t* = 0 ps, both transients show a monoexponential decay. Then, they start to substantially deviate from each other at *t* = 1000 ps. The transient of the 3D structure continues to follow the same monoexponential decay, whereas the planar structure response can be well described by a biexponential function. The decay time of the microrod structure was determined to (530 ± 30) ps (Fig. 5(a) black transient). For the planar structure, an initial decay time of (400 ± 30) ps and a longer time constant of (4.1 ± 0.5) ns were estimated (Fig. 5(a) red transient). Although the longer time constant component is hardly visible in the shown transient, it could be clearly observed in a transient recorded over a longer duration. The initial decay time of the planar structure is in the same order of magnitude as the decay time of the 3D LED, but the long component of the planar structure reveals a decay dynamic about ten times slower than the microrod sample.

Additional measurements on similar structures reveal that the excitation laser wavelength only has minor influence on the recombination dynamics over the 325 nm–375 nm range, for both planar and 3D structures (not shown here). The shape of the transients is the same when the InGaN is excited resonantly at a wavelength of 375 nm or using the 325 nm excitation wavelength, while the estimated initial decay times are 30 ps–50 ps shorter for the 375 nm excitation.

Time-resolved PL measurements were also performed at different excitation intensities. Figure 5(b) shows the decay time of the 3D microrod structure and the initial (fast) decay time of the planar structure for different energy densities. For both samples, a reduction of the decay time by about 30% is observed for about two orders of magnitude higher excitation intensities.

## Discussion

Figure 2 shows a blue shift of the InGaN luminescence peak with increasing excitation energy densities of the fs laser pulses. For low fs laser pulse energy densities in the range of 6⋅10^−6^ J/cm ^2^ to 47 ⋅ 10^−6^ J/cm^2^, the position of the InGaN luminescence peak coincides with the cw laser measurement. The blue shift observed at higher excitation energy densities can be explained by band-filling effects, favouring the recombination of electrons and holes from states higher in the energy bands. Additionally, for the planar structure with the polar c-plane MQW, the screening of the QCSE at higher excited charge carrier densities can lead to a further blue shift of the luminescence peak^15^. The GaN luminescence peak shows no shift because most charge carriers will be captured in the InGaN QWs which are only a small percentage of the whole structure. Therefore, in contrast to the GaN, the InGaN QWs are highly affected by the high excitation energy densities. Broadening of the high energy side of the InGaN luminescence band would also be expected, although this was not observable because the InGaN band is already relatively broad under the cw laser excitation - this is due to the inhomogeneous distribution of the indium in the MQW structure and the presence of localization centres. The broadening of the band is therefore less pronounced for the InGaN MQW sample. Furthermore, the high energy side of the InGaN band overlaps with the GaN luminescence band so that reabsorption processes in the different layers of the sample have to be taken into account. Such a shift of the luminescence without broadening was also observed by Wang *et al*.^15^ but under different excitation conditions. They used a cw laser, a smaller range of the excitation power densities and a different excitation wavelength. It has to be stressed that the energy shift of the InGaN luminescence band is reversible below the damage threshold of the samples.

An indium concentration of about 12% can be inferred by applying Vegard’s law for the InGaN emission peak of the 3D structure under cw excitation^16^. The indium composition is in the expected range considering the growth conditions used, despite not taking into account the effect of polarisation or dimensionality on the emission wavelength. The indium content of the planar sample was estimated to be about 14%, with the 2% difference between the two samples being attributed to the substantially different growth conditions on the m- and c-plane.

The results presented in Fig. 3 and Table 1 clearly demonstrate that the intensity ratio *r* of the (Al)GaN to the InGaN luminescence becomes larger for increasing excitation energy densities. This observation applies to the planar as well as to the 3D structures. The relative peak intensity of the InGaN, with respect to the (Al)GaN luminescence band, is strongest for the smallest energy densities used. The (Al)GaN intensity increases faster than the InGaN intensity as the energy density is increased, causing the spectrum to be dominated by the (Al)GaN luminescence peak. The most striking effect, is the irreversibility of changes in *r*(Φ) once an energy density threshold has been reached. Based on SEM images of the irradiated areas, the damage threshold is determined to be Φ = 0.05 J/cm^2^ for our samples (at 1 kHz, 100 fs, 325 nm). This is the value at which a surface modification in the SEM images was visible and the measurements performed for Table 1 showed a decrease in the PL intensity measured at 0.01 J/cm^2^ when the sample had been previously excited with energy densities larger than 0.05 J/cm^2^. The modification threshold might be slightly different for the various samples due to the different sample compositions and geometries but the experiments reveal that they are in the same order of magnitude.

**Figure 6:**
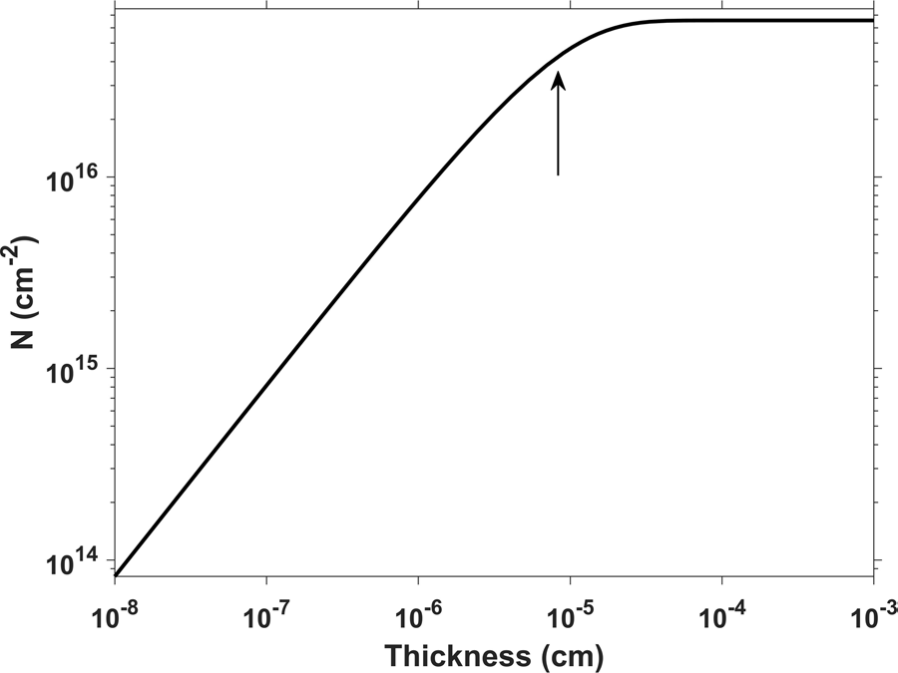
Calculated dependence of the excited charge carrier density *N* per area on the GaN layer thickness.

The excited charge carrier density at the observed damage threshold can be estimated using the model of ref.^17^, which assumes a set of rate equations in the linear optical regime. It has to be pointed out that, in our experiments, nonlinear effects (like multi-photon absorption) play a substantial role so that the model will yield a lower limit for the real excitation density. We assume a layer of GaN (i.e., a planar structure) and use the absorption coefficient of GaN *α* = 125 ⋅ 10^3^ cm^−1^ at 325 nm, the reflectivity *R* = 0.2, and the laser pulse energy density of 0.05 J/cm^2^. For the charge carrier density *N* per area, we obtain the following dependence on the layer thickness, assuming a laterally homogenous distribution of the carriers (Fig. 6).

The penetration depth for the 325 nm laser wavelength in GaN is about 80 nm which is indicated in the plot of Fig. 6 (marked with arrow). For a layer thickness of 100 nm, we get an excited charge carrier density of about 7 ⋅ 10^21^ cm^−3^. It is reasonable to assume that at such high carrier densities (considering nonlinear absorption effects, it would be even higher), material modifications like ablation or ultrafast melting occur. In comparison, we calculated the excited charge carrier density for the irradiation with the HeCd laser at a power density of 10 kW/cm^2^ which was, for example, used for the PL spectrum shown in Fig. 2(a). We used a rate equation from the literature^18,19^ and assumed the same absorption coefficient as above, a thickness of 100 nm and a decay time of 300 ps. Thereby, we get an excited charge carrier density in the order of 10^17^ cm^−3^ which is about four orders of magnitude smaller than for the estimated damage threshold.

Schneider showed in^12^ that the crystalline structure in a c-plane GaN wafer was permanently modified above a threshold of 0.24 J/cm^2^, using excitation with a pulse width of ∼120 fs, at a wavelength of 795 nm and a 1 kHz repetition rate (100 pulses applied per unit area). Our smaller threshold value is reasonable as the corresponding photon energy in our experiments is larger (3.81 eV compared to 1.56 eV in the previous experiments), in particular above the GaN band gap, and as the number of incident pulses is much higher.

The increase of the (Al)GaN to the InGaN luminescence intensity ration, *r*, by about two orders of magnitude with increasing laser fluence, as illustrated in Fig. 3, can be related to band filling effects and the modification of the structures. When increasing the excitation energy density, additional charge carriers are excited in the InGaN QWs, as well as in the GaN. At a certain point, most states in the QWs are occupied and even higher energy densities lead to a greater population of the GaN states and therefore to an increase in the intensity ratio *r*. By reaching the damage threshold, first the outermost layers including the QWs are destroyed, which further emphasises the GaN luminescence.

The time-resolved PL measurements with excitation power densities close to the damage threshold revealed a biexponential decay dynamic for the planar LED structure and a monoexponential one for the 3D structure. The biexponential transient can be explained by the quantum confined Stark effect. Internal piezoelectric fields cause he QCSE, resulting in band bending and a separation of electron and hole wave functions, reducing the recombination rate and increasing the decay time. Directly after the excitation, the high density of charge carriers screens the QCSE which results in the fast initial decay rate^6^. The QCSE plays a minor role in the 3D microrod structure because the quantum wells are mainly grown on the non-polar sidewalls, hence the monoexponentially decaying emission.

The decrease of the initial decay time with increasing pulse power (Fig. 5(b)) can be attributed to the high amount of excited charge carriers in the InGaN QWs, as seen in the results of Fig. 2^20^. The probability for the recombination process is higher if more charge carriers are excited. In the 2D structure the decay time at the beginning of the recombination process is further reduced because the higher charge carrier concentration shields the QCSE more strongly^21^.

Wrapping up, our results show that band-filling and screening of piezoelectric fields strongly influence the PL emission characteristics and recombination dynamics in 2D and 3D InGaN/GaN heterostructures at excitation power densities close to the damage threshold. Reliable and reproducible measurements of the recombination dynamics in the InGaN QWs are possible at least up to Φ = Φ_*damage*_/5. Above an energy density of 0.3 J/cm^2^, the ratio of the GaN to the InGaN luminescence intensity *r*(Φ = 0.01 J/cm^2^) starts to substantially and irreversibly increase. At the damage threshold of 0.05 J/cm^2^, modifications of the sample occur as contrast difference in the SEM images after 10 minutes of irradiation. In this energy density regime, reproducible measurements are still possible if just short excitation times are used. It has to be kept in mind that for femtosecond laser systems with low repetition rates (e.g. 1 kHz systems) and short pulse lengths of about 100 fs, the pulse power is roughly 10^10^ times larger than the measured average power which is in the range of a few tens of milliwatts. These high pulse powers or energy densities can easily damage the GaN heterostructures, which are typically considered to have good thermal and chemical stability^22^.

## Conclusion

In summary, the energy density dependence of the optical properties of 2D and 3D LED structures was studied using femtosecond laser pulses for time-integrated and time-resolved photoluminescence (PL) measurements. It has been found that the InGaN luminescence peak blue shifts by several tens of meV as the excitation energy density increases, due to band filling effects. For energy densities in the range of 6 ⋅ 10^−6^ J/cm^2^ to 47 ⋅ 10^−6^ J/cm^2^ the difference between the InGaN peak emission energy under excitation with the fs laser system and the cw laser vanishes. The damage threshold of InGaN/GaN heterostructures was determined to be about 0.05 J/cm^2^ for the excitation conditions used in this study. Close to the damage threshold, the intensity ratio of the (Al)GaN to the InGaN luminescence strongly changes and increases by 1–2 orders of magnitude as the energy density is raised from 1 ⋅ 10^−2^ J/cm^2^ to 3 ⋅ 10^−1^ J/cm^2^. Irreversible changes of the intensity ratio *r*(Φ = 0.01 J/cm ^2^) were found above an energy density of 3 ⋅ 10^−1^ J/cm^2^. On SEM images of a planar GaN structure, we found irreversible modifications for the damage threshold of 0.05 J/cm^2^ after 10 minutes of irradiation.

The time-resolved measurements demonstrate that the QCSE is clearly visible for the planar sample, but it only plays a minor role for the 3D structures. Furthermore, the decay time of the 3D and the initial one of the 2D structures decreased by about 30% when increasing the excitation energy density by about two orders of magnitude influenced by band filling effects and shielding of the QCSE. This additionally shows the importance of choosing the appropriate energy density for the structures under investigation to get reliable measurements without irreversible modifications of the samples.
